# Pain Management Throughout Pediatric Laparoscopic Appendectomy: A Systematic Review

**DOI:** 10.7759/cureus.49581

**Published:** 2023-11-28

**Authors:** Ethan Slouha, Brandon Krumbach, Jheanelle A Gregory, Stefan J Biput, Allison Shay, Vasavi R Gorantla

**Affiliations:** 1 Anatomical Sciences, St. George's University School of Medicine, St. George's, GRD; 2 Anatomical Sciences, St. George’s University School of Medicine, St. George's, GRD; 3 Biomedical Sciences, West Virginia School of Osteopathic Medicine, Lewisburg, USA

**Keywords:** laparoscopic appendectomy, nerve blocks, narcotics, pain management, pediatric surgery, analgesia

## Abstract

Opioid-related fatalities are a leading cause of accidental death in the United States. Appendicitis is a common cause of abdominal pain in children and adolescents. The management of pain throughout the laparoscopic appendectomy (LA) in the pediatric population is a critical concern. This study aimed to evaluate trends in analgesic use and patient satisfaction following LA, with a focus on reducing the reliance on opioids for pain management. From 2003 to 2023, 18258 articles were filtered for all types of analgesic use with LA. The publications were screened using Preferred Reporting Items for Systematic Reviews and Meta-Analyses (PRISMA) guidelines, and 19 studies were included for analysis and review. The study included peer-reviewed experimental and observational studies involving individuals under 18 years. Pain management strategies varied across studies, involving a combination of analgesics, nerve blocks, and wound infiltrations. Analgesics such as acetaminophen, non-steroidal anti-inflammatory drugs (NSAIDs), and opioids were administered before and after surgery. Some studies implemented patient-controlled analgesia (PCA) pumps. Other studies explored non-pharmacological interventions like magnetic acupuncture. The results showed a reduction in the need for postoperative analgesics in patients treated with LA, particularly when using non-opioid medications and novel analgesic techniques. Pediatric patients who received gabapentin reported lower opioid use, shorter hospital stays, and high satisfaction rates. However, the reliance on opioids remained significant in some cases, particularly among patients with peritonitis who required more morphine. Pain management in pediatric patients is multifaceted, involving preoperative and postoperative analgesics, nerve blocks, and PCA pumps. Efforts to improve pain management following pediatric LA while reducing opioid reliance are essential in the context of the ongoing opioid epidemic. The findings from this study highlight the potential benefits of non-opioid analgesics, nerve blocks, and alternative methods for managing postoperative pain in <18 appendectomy patients. Further research and standardization of pain management protocols are needed to ensure optimal patient outcomes and minimize the risk of opioid-related complications.

## Introduction and background

Opioid epidemic

Accidental deaths are the fourth highest cause of death, and the leading cause of accidental death is drug overdose in the United States [1]. The main drug of choice in these accidental deaths is opioids [1]. In 1803, morphine was extracted from opium and changed how pain control was managed in modern medicine [2]. It was not until later in the 19th century that German physician Dr. Eduard Livenstein accurately described opiate addiction [2]. Seventy-one percent of opioid-related deaths occur in the 25-34 years of age group, with most victims being males [1]. Caucasians, Alaskan Natives, and Native Americans are the most at risk [1].

First-time abusers increased from 628,000 in 1990 to 2.4 million in 2004, while emergency room visits involving opiate abuse increased by 45% from 2000 to 2002 [2]. Admission for the treatment of prescription opioids increased by 400% between 1998 and 2008 [3]. There’s been a significant overprescription of opioids between the physicians who prescribe them and the allowance by the pharmaceutical companies to do so [1]. The link between the medical necessity of opioids and their abuse potential is now heavily being investigated [2]. There’s a significant clinical dilemma as the appropriate use of opioids can be required to treat certain levels of pain for optimal functioning, however, overuse and misuse lead to significantly drastic personal and societal consequences [3]. A challenge remains reducing abuse and adverse effect potential while not creating a barrier to patients who legitimately rely on opioids for medical interventions [2].

Appendicitis

Appendicitis is the inflammation of the appendix and most commonly stems from appendix blockage, often due to various factors such as fecal matter, undigested food, calcified deposits like appendicoliths, or lymphoid hyperplasia [4,5]. This ultimately leads to abdominal pain near the navel and thickening of the appendix wall [4]. There are up to 250,000 cases of reported appendicitis annually, the majority of patients between 10 and 19 years of age are diagnosed in the emergency department [5,6]. Common signs and symptoms are anorexia, fever, vomiting, periumbilical pain, migrating pain to the right lower quadrant with tenderness, and pain with movement [4]. Guarding in the lower right abdomen suggests parietal peritoneum irritation, while widespread guarding indicates a case of severe appendicitis [5,7].

The pediatric appendicitis score (PAS) is a 10-point scale that uses medical history, physical examination, and lab findings to assess appendicitis risk in children with abdominal pain [4]. A PAS score of ≤3 indicates a low risk for appendicitis and permits discharge home with instructions [4]. A score of ≥7 or 8 indicates a high risk, necessitating surgical consultation or prompt imaging per local protocols [4]. A score of 3-6 or 7 is intermediate, with options including surgical consultation, diagnostic imaging, hospital observation with repeated examinations, or a combination [4]. Appendicitis can be further divided into complicated or uncomplicated. Uncomplicated is inflammation without phlegmon, gangrene, purulent fluid, or an abscess, while complicated involves periappendiceal phlegmon with or without perforation, gangrene, or a perityphlitis abscess [7]. Appendicitis in kids is treated in two different ways, one being antibiotics and the other, which is more definitive, is surgery.

Appendectomy

The appendectomy is the gold standard of treatment for appendicitis as it is also the only definitive treatment [8]. There are two ways an appendectomy can be performed: open appendectomy (OA) or laparoscopic appendectomy (LA); however, before surgery, prophylactic antibiotic therapy is started to prevent further infection [8]. In 1895, Dr. McBurney described the surgical management of appendicitis in which he denounced the method of a midline incision and proposed the McBurney’s incision, which is located 1/3rd of the way from the anterior-superior iliac spine, following Langer lines to the umbilicus [8]. This incision is usually followed by an Elliot or Rockey-Davis transverse incision next to McBurney, leading to the medial side of the rectus abdominis [8]. The LA uses the periumbilical Hasson technique to create short incisions around the right lower quadrant of the abdomen, and then a Versess needle or an optical trocar is used to create a pneumoperitoneum [8]. An optical trocar is placed in the right lower quadrant just above the pubis, and another is placed in the left lower quadrant to provide visual aid [8]. The mesoappendix is dissected in both procedures, and the appendiceal vessels are divided [8]. LA has been preferred over OA as it is associated with a lower risk of infection, decreased hospital stay, better cosmetic results for patients, and decreased prescription of analgesics [9,10].

Aim

There’s a significant increase in opioid use disorder, accounting for the majority of drug overdoses, throughout the globe, and the majority of cases arise from prescription opioids. This occurs in younger individuals at a higher rate, emphasizing the need to better control opioid dispensement in adolescents undergoing significant pain. As technology advances, there are new techniques to reduce the pain and extensiveness of previous forms of treatment, such as OA versus LA. This study aimed to evaluate the analgesic trends following LA and if there is a need for opioid-based treatment before, during, and after treatment while maintaining optimal patient satisfaction levels.

## Review

Methods

A meticulous literature search was implemented from January 1, 2003, to December 31, 2023, using three catalogs: PubMed, ProQuest, and ScienceDirect. The keywords used to search for the articles were "analgesics and laparoscopic appendectomy" and "pain management and laparoscopic appendectomy." The search was oriented around peer-reviewed experimental and observational publications on individuals <18 years of age. Publications published prior to 2003, not written in English, and duplicates were excluded from the eligibility review. Once publications were attained, four independent co-authors evaluated the information from the publications and compiled and compared the results. These publications were evaluated based on the title, abstract, study type, age of participants, and full-text accessibility. The preliminary analysis of the three databases resulted in 18,258 publications. Keyword specifics, age range, and information gathered from the abstract further eliminated papers. A total of 19 publications were found that covered the intent of this review according to the below criteria.

Inclusion Criteria

The criteria for inclusion increased publications that focused on all types of analgesics used with LA, were published between 2003 and 2023, were peer-reviewed observational or experimental studies conducted on humans, written in English, and full-text.

Exclusion Criteria

**Figure 1:**
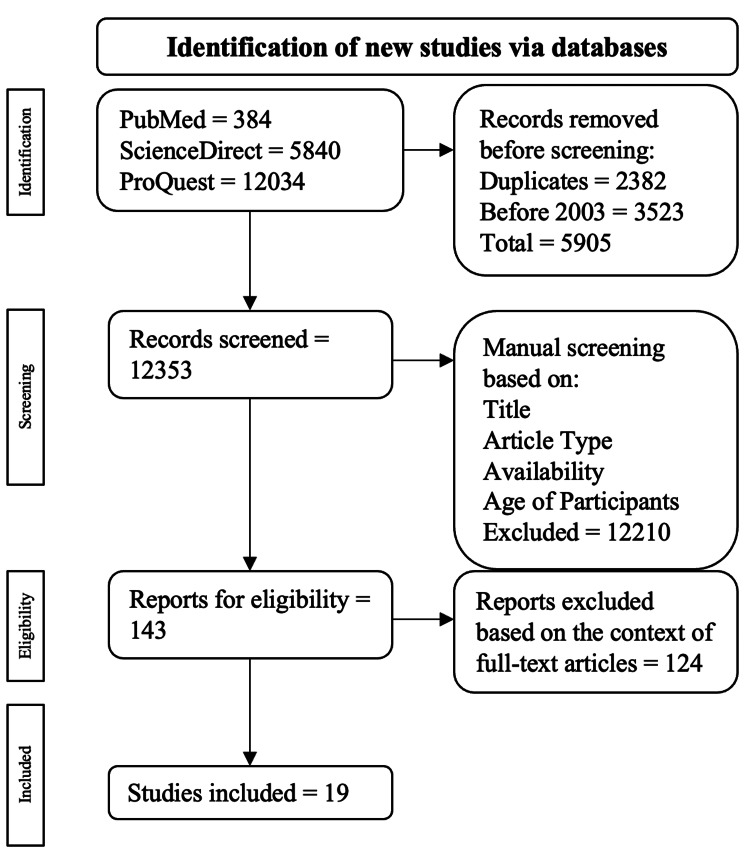
Visual representation of the inclusion and exclusion criteria process. The pathway used for the inclusion and exclusion criteria came from the PRISMA review [11]. PRISMA: Preferred Reporting Items for Systematic Reviews and Meta-Analyses

The criteria for exclusion consisted of eliminating meta-analyses, case reports/series, and narrative reviews. This publication's inclusion and exclusion methods are drawn out in Figure 1.

Bias

Each publication included in this study was individually assessed for bias. The overall bias in this publication is minimal when viewing both experimental and observation study standards. The Grading of Recommendations, Assessment, Development, and Evaluations (GRADE) scale was the specific bias tool used to evaluate the publications. The GRADE tool evaluates the risk of bias by assessing each article's imprecision, indirectness, and publications.

Results

A total of 18,258 publications were found as follows: 384 were from PubMed, 5840 were from ScienceDirect, and 12,034 were from ProQuest. Among the exclusions, 2382 were duplicates, and 3523 were published before 2003. This resulted in 5905 publications being excluded during the automatic selection process, leading to 12,353 publications for manual selection. Publications were manually assessed through the previously mentioned criteria, resulting in 143 articles being checked for eligibility through full-text analysis. Ultimately, 19 articles were used.

The basis of pain management throughout the appendectomy was relatively standard with anesthesia induction, however, some studies implemented different anesthetics in the form of wound infiltration and nerve blocks. The use of lidocaine and bupivacaine combined with dexmedetomidine for wound infiltration has been shown to significantly decrease pain scores following surgery. With transabdominal plane (TAP) blocks, bupivacaine showed a significant decrease in pain postoperatively. It delayed the need for a rescue dose of any analgesia and the total amount of opioid use compared to ropivacaine. Regarding specifically postoperative management of pain, parents and patients expressed the same amount of satisfaction concerning non-opioid-based prescriptions compared to opioid-based prescriptions. Some surgeons still choose to use PCA pumps, however, some studies observed that more non-opioid analgesics are required for a longer duration once the patient is removed from the PCA pump. One alternative drug used to try and reduce postoperative pain was gabapentin, which was found to be successful at reducing pain and the need for a rescue opioid. Lastly, one study evaluated a non-pharmacological approach, magnetic acupuncture, and found some success in reducing patient's pain following the procedure. All studies used in this review can be found in Table 1.

**Table 1:** Summary of articles used to create the data of the systemic review via PRISMA guidelines [11]. TAP: transabdominal plane; PCA: patient-controlled analgesia; IVA: intravenous analgesia; LA: laparoscopic appendectomy; NSAIDs: non-steroidal anti-inflammatory drugs; VAS: visual analog scale

S.no.	Author	Country	Design and study population	Findings	Conclusion
1	Ferguson et al. (2020) [12]	USA	Retrospective cohort study, (n = 546)	28% of pediatric patients received opioids postoperatively, which was associated with longer preadmission symptom duration, had a higher chance of having complicated appendicitis, and were more likely to have received opioids preoperatively. All pediatric patients received opioids during their operation. For every preop morphine, milliequivalents (MMEs) per kg received preoperation was associated with a further 0.29 MMEs per kg postoperation.	Giving opioids preoperatively was independently associated with a further increase in postoperative use in pediatric patients who underwent laparoscopic appendectomy. As a result, to reduce postoperative opioid use in pediatric patients, alternatives to preop opioids must be created.
2	Hamil et al. (2015) [13]	New Zealand	Experimental study (n = 130)	Pediatric patients who underwent the nerve block had reduced global pain scores compared to the control group in the first 3 hours postoperatively, however, the groups did not have differences between opiate usage, length of stay, or recovery.	Bupivacaine plus adrenaline as a rectal sheath block did prove to reduce postoperative pain in 8-14 years old pediatric patients who underwent laparoscopic appendectomy. This study suggests that a rectus sheath block can be incorporated into a multi-modal recovery program.
3	Sandeman et al. (2011) [14]	Australia	Randomized control trial (n = 93)	There was no significant difference in the proportion of pediatric patients using more than 200 mcg/kg of morphine between the two groups. There was no significant difference in morphine use, time until first use, or amounts of use of other analgesics like paracetamol between the two groups.	TAP blocks were responsible for increasing anesthesia time by 14 min on average. Still, no significant evidence was found to show the benefit of TAP over local anesthetic at port site infiltration when considering postoperative pain measurement.
4	Sola et al. (2019) [15]	USA	Randomized control trial (n = 80)	The time to transition from PCA to oral pain medications was not significantly different between pediatric patients with IVA and those without IVA. Pediatric patients in the IVA group had similar intravenous opioids and pain scores compared to the non-IVA group. The amount of oral opioids also showed no significant difference between the groups.	Pediatric patients receiving IVA had similar transition times from PCA to oral pain medications after laparoscopic appendectomy for perforated appendicitis, with no changes in their opioid use or pain perceptions.
5	Baird et al. (2019) [16]	Canada	Randomized control trial (n = 116)	No notable distinctions were observed among the intervention, placebo, and baseline groups. The overall in-patient morphine consumption showed no significant differences between randomized groups or between ropivacaine and baseline groups. While operative times were similar across groups, nebulization hindered visualization for 63% of surgeons.	Nebulized ropivacaine did not result in a decrease in postoperative morphine usage or pain scores following laparoscopic appendectomy for uncomplicated appendicitis in pediatric patients. It negatively impacted visualization during surgery and likely increased cost; using nebulized intraperitoneal analgesia was unnecessary.
6	Both et al. (2018) [17]	Switzerland	Retrospective cohort study (n = 116)	None of the pediatric patients receiving lidocaine displayed any indications of systemic toxicity, neurological issues, or circulatory problems. There was a non-significant variance in emergence delirium.	The study found no adverse consequences in pediatric patients who received intravenous (IV) lidocaine during laparoscopic appendectomy under general anesthesia.
7	Elnabtity and Ibrahim (2018) [18]	Egypt	Randomized control trial (n = 52)	VAS scores were lower in group bupivacaine + dexmedetomidine (BD) than in group B (bupivacaine). Group BD also had a longer time for rescue analgesia, less analgesic needed, shorter hospital stay, and higher parent satisfaction.	This study showed a statistically significant increase in pain management success when adding dexmedetomidine to bupivacaine intraperitoneally compared to using just bupivacaine alone.
8	Kaszynski et al. (2021) [19]	Poland	Experimental study (n = 71)	There were no significant differences in cumulative nalbuphine doses within the first 24 hours postextubation. The lidocaine group had lower intraoperative fentanyl use and a longer time until the first analgesic request.	Intraoperative lidocaine reduced opioid needs during laparoscopic appendectomy in pediatric patients, but this effect was short-lived and did not impact opioid use within the first 24 hours after stopping lidocaine infusion.
9	Feng et al. (2023) [20]	Germany	Experimental study (n = 102)	Group 1 (standard analgesic pain medication plus magnetic acupuncture) had the least usage of acetaminophen and ibuprofen and opioid usage compared to group 2 (standard analgesic pain medication plus placebo acupuncture using non-magnetic metal disks) and group 3 (standardized pain medication alone) and overall reported a high satisfaction when assessing magnetic acupuncture.	Using magnetic acupuncture led to a cumulative decrease in the use of analgesics and a higher satisfaction rating compared to the other group. The paper states that magnetic acupuncture is safe and effective, however, further research is needed.
10	Liu et al. (2013) [21]	USA	Retrospective cohort study (n = 206)	The incidence of substantial pain post appendectomy was reported at 12% when multimodal pain management was used. This was statistically significant and lower than earlier reports using unimodal analgesia. Pediatric patients who had complications of peritonitis recorded more pain, higher use of opioids, and an unmet need while using PCA, with higher documentation of respiratory depression than those with simple appendicitis.	The use of multimodal pain management (local anesthetic infiltration, opioid by PCA, NSAIDs, and oral acetaminophen/hydrocodone) showed a statistically significant decrease in severe pain. The author notes that more studies are needed for pediatric patients with minimal opioid requirements and the ability to modify this regimen for maximum satisfaction of pain control.
11	Baxter et al. (2018) [22]	USA	Retrospective cohort study (n = 87)	The gabapentin (GP) group required significantly fewer postoperative opioids (0.034 mg morphine equivalents (ME)/kg) compared to the non-gabapentin (NG) group (0.106 ME/kg). Both groups had similar timeframes to reach pain scores ≤3.	Total postoperative use of opioids is significantly reduced with the use of gabapentin in pediatric patients undergoing appendectomy.
12	Maloney et al. (2018) [23]	USA	Retrospective cohort study (n = 275)	Pediatric patients who underwent pre incision rectus sheath block had significantly lower opioid usage compared to those who had conventional local anesthetic infiltration. Pediatric patients also reported lower initial mean and lower mean pain scores using the rectus sheath block, and the time for rescue analgesia was longer for pediatric patients with the rescue sheath block.	For pediatric patients who underwent single-incision transumbilical laparoscopic-assisted appendectomy, pre incision rectus sheath block was proven to have decreased opioid consumption and lower pain scores compared to local anesthetic infiltration. Rectus sheath block is, therefore, a more efficient way to control pain postoperatively than local anesthetic infiltration.
13	Visoiu et al. (2021) [24]	USA	Experimental study (n = 50)	The median duration of periumbilical numbness did not significantly differ between the two study groups. No significant difference was noted in periop analgesic consumption, pain and anxiety scores, sedation, or hemodynamic instability.	The addition of clonidine did not prolong the rectus sheath nerve block length but was tolerated well in pediatric patients.
14	Alkhoury et al. (2014) [25]	USA	Prospective cohort study (n = 207)	Non-opioid medication was found to be at least as effective as opioid-based therapy for postoperative pain control. Both groups had equivalent medication days and similar times of return to normal activity. 97% of the parents of pediatric patients in the non-opioid group stated that the pain was controlled by the prescribed medication, compared to 90% in the opioid group.	After a non-complicated pediatric laparoscopic appendectomy, non-opioid was found to be equivalent to opioid-based therapy for outpatient oral analgesia, with higher parenteral satisfaction.
15	Freedman-Weiss et al. (2019) [26]	USA	Retrospective cohort study, (n = 336)	This study analyzed patients aged 7-20 years who underwent uncomplicated laparoscopic appendectomies between January 2016 and August 2017. The amount of opioids prescribed postop was measured and converted to oral morphine equivalents (OMEs). Of the 336 patients, 148 were operated on by general surgeons, and 188 were operated on by pediatric surgeons. It was found that pediatric surgeons administered fewer opioids compared to general surgeons.	General surgeons prescribe more opioids to adolescent patients compared to pediatric surgeons, and as a result, this has contributed to the opioid epidemic. Therefore, increased education and guidelines are needed to regulate the prescription of opioids given to pediatric and adolescent populations.
16	Freedman-Weiss et al. (2020) [27]	USA	Retrospective cohort study (n = 73)	This study analyzed 73 patients aged 5-20 years who underwent laparoscopic appendectomy during the period. This study was set out to determine the postoperative opioid needs in pediatric and young adult patients after their procedure. All opioids were converted into morphine milligram equivalents (MME). It was found that 83% of the sample did not require an opioid and reported pain <4/10 after being discharged, using the Defense and Veterans Pain Rating Scale. 5 patients required an opioid after being discharged, averaging an MME of 23 (3 x 5 mg oxycodone).	Based on the study done, most patients have comfortable pain levels and adequate analgesia without the use of opioids. As a result, the prescription of opioids should be regulated to not exceed 25 MME as a method to prevent the opioid epidemic.
17	Manworren et al. (2016) [28]	USA	Retrospective cohort study (n = 200)	It was found that pediatric patients who were administered perioperative IV ketorolac and opioids had significantly lower pain levels during the first 24 hours postoperative and also had lesser morphine equivalents of postoperative opioids during the first 24 hours postoperation.	This study was done to demonstrate the benefits and advantages of having a multi-modal analgesic approach for pediatric patients who underwent laparoscopic appendectomy.
18	Phillips et al. (2005) [29]	Canada	Retrospective cohort study (n = 342)	Operating time was significantly decreased in both acute appendicitis and perforated appendicitis. Anesthetic time was also decreased in both LA and perforated appendectomy. Postoperative abscess formation for perforated appendectomy decreased from 36.2 to 16.5%. No change was recorded in the analgesia used.	Over a 10-year period, there was a significant decrease in time for anesthesia, operating times, abscess formation, and conversion rates.
19	Lascano et al. (2023) [30]	USA	Retrospective cohort study (n = 29,467)	Pediatric patients who had received gabapentin for pain management in LA had a significantly decreased opiate use postoperatively, as well as reduced days on opioids.	Gabapentin should be used more frequently to reduce opioid use and assist in pain management following LA.

Discussion

Pain Management Throughout the Appendectomy

Upon admission to the ER, physicians attempt to control the pain before any tests begin to determine if the child does have appendicitis. However, once appendicitis is diagnosed, the medication regimen changes to prepare the child for surgery. First, pain medications such as acetaminophen at 15 mg/kg and IV opioids such as morphine and fentanyl are given [12-15]. Up to 0.3 mg/kg of morphine or up to 2 mcg/kg of fentanyl are administered, but the specific pain medication varies based on the provider’s preference [13]. The preoperative drugs are typically administered more than 1 hour before the surgery begins and are usually paired with an anti-emetic to prevent nausea from the pain medications [12,13].

After administering pain medications, the child gets a standard midazolam premedication at 0.5 mg/kg intravenously, orally, or rectally, about 30 minutes before moving to the operating room [16-19]. Pediatric patients are more likely to be sedated through general anesthesia before numbing the surgical site via infiltration or nerve block. Anesthesia was induced by a mixture of propofol, rocuronium, sufentanil, and fentanyl and was maintained using sevoflurane and fentanyl if hypertension was present [14-21]. Some anesthesiologists switched sufentanil with alfentanil or rocuronium with atracurium, but every other anesthetic remained the same [17,19]. Some studies used an additional bolus of lidocaine for the anesthesia to test the analgesic effects following surgery and the need for pain medication postoperation [17,19]. One study also added nebulized ropivacaine on top of traditional general anesthesia to see if it would reduce the morphine a child would need postoperatively [16].

There are two common ways the child's incision site and location can be anesthetized as follows: wound infiltration or a newer method for the procedure, a nerve block. One percent lidocaine and 0.25% bupivacaine were the only two anesthetics found to be used for wound infiltration, but the dosings were not standardized [12,15,17,18,20-23]. Most nerve blocks observed through the articles were TAP blocks, which required ultrasound to guide the incision [12,13,23]. This technique is usually done with sodium channel blockers, and 0.25% bupivacaine and 0.2-0.5% ropivacaine were the most commonly used ones in combination with adrenaline or clonidine [14,23,24]. Doses for both bupivacaine and ropivacaine were not standardized between studies. The only other block mentioned was a quadratus lumborum block, but the type of anesthesia was not described [12].

Along with anesthesia administration during surgery, pain medications were given concurrently. Some pediatric patients received IV acetaminophen or non-steroidal anti-inflammatory drugs (NSAIDs) after endotracheal tube placement and midazolam [19]. Other pediatric patients, however, were given opioids such as 2 mcg/kg fentanyl or 0.1 mg/kg morphine, and this was based on the child’s response to surgical stimuli [12,14,15,17,21]. Dexmedetomidine was often added to prevent the potential emergence of delirium or agitation [15]. Ultimately, the attending anesthesiologist decided to add additional pain medications [23]. Before the anesthesia reversal, the anesthesiologist administers IV acetaminophen or NSAID, sometimes with dexamethasone [16,24]. One study reported that all pediatric patients received 1 mcg/kg and up to 4 mcg/kg of fentanyl at the end of the procedure, but no other studies reported this [14].

Following surgery, the anesthesiologist would repeat the wound infiltration using 0.25% bupivacaine, but this only seemed to be the case in studies that chose wound infiltration as their first form of anesthetizing the wound site [17,18,20]. Both et al. continued the lidocaine infusion until the child was transferred to the recovery wing [17]. To reduce the muscle relaxant effect, neostigmine and atropine were administered [18]. Further management of pain required pain medications and was begun immediately after surgery. Some anesthesiologists start a basal infusion of morphine, while others aren’t administered any drugs until > 2 hours after surgery [12,21]. Typically, acetaminophen and NSAIDs are given regularly via IV in 6-hour intervals, and opioids are used as rescue medications [13,15,17-19,21,24,25]. Some pediatric patients start with NSAIDs and are transitioned to acetaminophen [15]. When opioids were needed, it was usually pethidine 1 mg/kg, 1 mg/kg of codeine, 0.1 mg/kg oxycodone, 0.05 mg/kg morphine, or 5 mcg/kg of hydromorphone via IV [16,18,19,21,24-29].

Some anesthesiologists have a protocol set up where the child is on a patient-controlled analgesics (PCAs) pump and basal morphine dose with varying doses of morphine depending on the anesthesiologist or purpose of the study [14,15,21]. No additional pain medications were given if the PCA was in use, and PCA was discontinued when the child regained bowel functions and could tolerate oral intake [14,15]. Once oral intake was tolerated, pediatric patients were usually switched to an acetaminophen and opioid mix every 4 hours for the next 24 hours [15,17,21]. One study gave gabapentin 10.1 mg/kg/day orally as their baseline pain medication to determine how well it controls postoperative pain and was combined with ketorolac in most pediatric patients [22]. Freedman-Weiss et al. observed that pediatric surgeons administered oxycodone significantly less than general surgeons [26].

There was one study that evaluated a non-pharmacological pain management approach through the use of magnetic acupuncture. All three following groups used standardized analgesia: acetaminophen, NSAID, and piritramide [20]. Ten specialized magnets were placed around the surgical site where acupuncture is promising, following the extubation and dressing of the umbilical wound [20]. The control group had similar-looking metallic disks that weighed the same, but everything else was similar [20]. They observed whether pain was alleviated [20].

Outcomes of Pain Management

Since the implementation of the LA, there has been a reduced need for postoperative analgesics and a decreased time between enteral eating and ambulation [29]. However, postoperative analgesics have not been reduced due to the physicians' preference [29]. Recently, there have been changes in certain drugs used in the induction of general anesthesia. One study decided to observe the effects of nebulized ropivacaine in addition to traditional anesthesia. The nebulized ropivacaine did not alter the postoperative pain score and morphine usage compared to the control, and there was no change in days to ambulation or length of stay in the hospital [16]. One thing to note with ropivacaine is that while operative times were similar to control, the nebulization obscured the visualization [16].

There have also been changes and evaluations of different anesthetics and their effects following wound infiltration in immediate and long-term perspectives concerning pain and the need for more analgesia. Bupivacaine combined with dexmedetomidine has been evaluated in its efficacy, and there were no differences recorded in the need for intraoperative fentanyl consumption but significantly increased time between surgery and needing first analgesia [18]. It led to significantly lower visual analog scale (VAS) pain scores concerning abdomen and shoulder pain, correlating with a decreased need for analgesia overall [18]. Pediatric patients had a significantly reduced hospital stay and a higher parent satisfaction rate in the bupivacaine and dexmedetomidine group [18]. Side effects such as perioperative bradycardia, nausea, vomiting, and dizziness were decreased when dexmedetomidine was added [18].

Lidocaine has also been evaluated in its use and effects on wound infiltration. The amount of sevoflurane consumed between pediatric patients with lidocaine and the control group did not increase, but there was a significant decrease in fentanyl intraoperatively [19]. Those who received lidocaine had a significantly longer time until they first requested analgesics and significantly fewer non-opioid and opioid but not partial opioid analgesics postoperatively [17,19]. Following treatment, side effects such as nausea were decreased, and there were no reports of cardio-respiratory collapse/arrest, refractory hypotension, bradycardia, anaphylaxis, systemic toxicity, or neurological complications [17,19]. Both et al. also observed a tendency towards a reduced occurrence of emergence delirium postanesthesia in the lidocaine group [17].

Another way to anesthetize the incision site is through a TAP block; studies compared TAP block to wound infiltration, and one study compared the addition of clonidine to the ropivacaine TAP block. When clonidine was added to the ropivacaine TAP block, the median duration of numbness was more significant [24]. One study observed that the use of bupivacaine led to a significant decrease in opioids intraoperatively compared to wound infiltration, an increase in IV acetaminophen, and no changes in IV NSAID use [23]. However, this was not observed with ropivacaine, whose pediatric patients used the same amount of opioids [14]. Immediately following surgery, both pediatric patients receiving bupivacaine and ropivacaine had significantly reduced VAS pain scores compared to local infiltration, however, this did not continue with the remaining time intervals in pediatric patients who receive ropivacaine [13,14,23]. Compared to using ropivacaine, bupivacaine showed a significant delay in pediatric patients needing a rescue dose of any analgesia and the total amount of opioid use [23]. Studies evaluating ropivacaine observed that with ropivacaine, there was no change in time for first opioid use and no difference in the need for supplemental non-PCA analgesic use, even when combined with clonidine [13,14,24]. Despite the difference between the two anesthetics, compared to wound infiltration, there was no difference in the overall child satisfaction, quality of life, or time taken to return to normal activities [13,14,23,24].

In studies just focusing on the use of pain medication, most studies have observed that non-opioids are at least equivalent to opioids concerning pain management when opioids were limited [15,25,27,28]. Results show even higher parental satisfaction in pediatric patients who received non-opioids [25]. When preoperative IV NSAIDs were used, they observed significantly lower mean pain intensity and pediatric patients received significantly lower morphine equivalents during the first 24-hour postoperative period [28]. Those who needed continued analgesics following discharge were small and were only prescribed three pills of oxycodone over three days [27]. However, Ferguson et al. observed that the administration of postoperative opioids was commonly associated with preoperative acetaminophen and morphine [12]. Pediatric patients who did not receive any preoperative opioids had a higher chance of not receiving any postoperative opioids [12]. Side effects include nausea, vomiting, and pruritus, with a higher incidence of respiratory depression [21,28].

Some physicians also decided to use PCA pumps for analgesia following surgery. When paired with IV acetaminophen, those with and without acetaminophen exhibited similar levels of pain score and amount of IV opioid pain medications [15]. Liu et al. found that PCA use correlates with peritonitis with appendicitis and that pediatric patients with peritonitis needed more rescue morphine [21]. Using a PCA pump significantly decreased pain rating compared to the PRN morphine group [21]. No significant difference was observed between the duration of PCA use and the time it took for pediatric patients to switch from PCA to oral pain medications for pediatric patients with and without IV acetaminophen use [15]. Individuals on PCA-alone required more oral acetaminophen once oral medications were added [15].

Two studies observed the impact of adding gabapentin in addition to the current pain management protocol of appendectomy. In addition to standard therapy, gabapentin should have similar operation timeframes to reach a pain score <4, along with reduced total amounts of intraoperative opioids [22]. Pediatric patients who were given gabapentin were less likely to be prescribed opioids, noted lower opioid use, short days of opioid use, and shorter duration of hospital stay [22,30]. No adverse effects of gabapentin were documented overall, as well as decreased revisit rates to the emergency department, length of stay, readmission rates within 30 days, and revisits due to surgical pain [22]. Feng et al. performed a holistic approach to pain management following LA through magnetic acupuncture. They found that not only there was a decrease in VAS pain scores, but they also had a decrease in medications and a satisfaction score of 9/10 16 hours postoperation [20].

Limitations

Only one-to-two studies covered each type of anesthesia/analgesia used, which limited a complete analysis of the specific treatment. However, it paints a picture of the varying protocols for treating appendectomies, which was the goal. It would have been beneficial to see trends in analgesic use in more than one study, as many publications report a decrease in analgesics. Gabapentin's use for LAs shows congruent results and would specifically have been great to get more information on, as this would highlight a good transition.

## Conclusions

Opioid abuse is a worldwide epidemic, with more and more individuals falling into the cycle of abuse. One of the terrifying side effects of opioid abuse is death. More recently, this has occurred at younger ages as adolescents are prescribed opioids for injuries and procedures. One of the procedures that used to require a relatively high dose of opioids was appendectomies. The appendectomy is termed America's first surgery and initially consisted of an open procedure where an incision was made at McBurney’s point, and the appendix was removed. LA was invented to allow one to three port sites compared to cutting open the abdomen. With this came shorter recovery time, decreased wound infection, and ideally decreased pain medications needing to be prescribed. Since the introduction of LA, there has been a decrease in the need for postoperative analgesics and a decreased time between enteral eating and ambulation. Still, some physicians have not reduced their prescribing rate of opioids. There have been trials of new ways to minimize morphine consumption, and while most were successful, some weren’t. Using bupivacaine plus dexmedetomidine and lidocaine for wound infiltration led to a significantly decreased amount of opioid use intraoperatively and a delay in time for first requesting pain management and the amount needed. TAP nerve blocks with ropivacaine or bupivacaine not hold a significant decrease in opioid use intraoperatively but a delay in the need for pain management and reduction in opioids, even more than wound infiltrations. A trend was that pediatric patients who did not receive opioids preoperatively were less likely to be given opioids postoperatively.

Due to the rapidly increasing opioid epidemic, it is time to look at the origin of it, which usually stems from physician prescriptions. Because the opioid epidemic occurs in adolescence, looking at the pain management of LA is essential as this is one way this population becomes addicted to opioids. The goal of gathering all the publications that focus on pain management for LA is to show that non-opioids are just as successful as opioids, and there are ways to make it so that there’s even a reduced use of non-opioid pain management. More research should be focused on not only trying to eliminate the need for opioids following LA but also educating physicians about this information to encourage a reduction in their prescription rate. While this won’t necessarily solve the opioid epidemic, it is still a decent contribution targeted at the population where opioid abuse starts.
